# An egocentric straight-ahead bias in primate’s vision

**DOI:** 10.1007/s00429-021-02314-8

**Published:** 2021-06-13

**Authors:** Benoit R. Cottereau, Yves Trotter, Jean-Baptiste Durand

**Affiliations:** 1 Centre de Recherche Cerveau Et Cognition, Université de Toulouse, 31052 Toulouse, France; 2 Centre National de La Recherche Scientifique, 31055 Toulouse, France

**Keywords:** Straight-ahead, Visual cortex, Gaze, Gain fields, Attention

## Abstract

As we plan to reach or manipulate objects, we generally orient our body so as to face them. Other objects occupying the same portion of space will likely represent potential obstacles for the intended action. Thus, either as targets or as obstacles, the objects located straight in front of us are often endowed with a special behavioral status. Here, we review a set of recent electrophysiological, imaging and behavioral studies bringing converging evidence that the objects which lie straight-ahead are subject to privileged visual processing. More precisely, these works collectively demonstrate that when gaze steers central vision away from the straight-ahead direction, the latter is still prioritized in peripheral vision. Straight-ahead objects evoke (1) stronger neuronal responses in macaque peripheral V1 neurons, (2) stronger EEG and fMRI activations across the human visual cortex and (3) faster reactive hand and eye movements. Here, we discuss the functional implications and underlying mechanisms behind this phenomenon. Notably, we propose that it can be considered as a new type of visuospatial attentional mechanism, distinct from the previously documented classes of endogenous and exogenous attention.

## Introduction

**Fig. 1:**
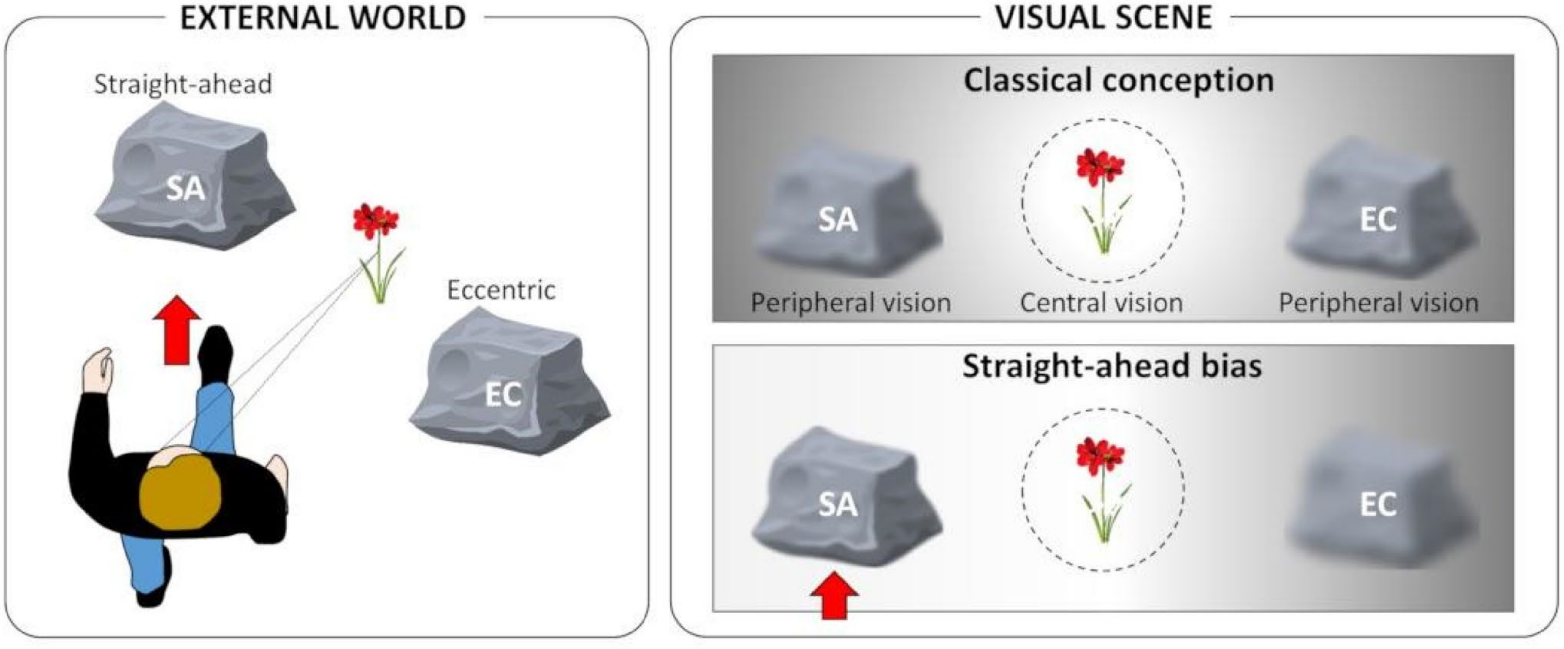
Privileged visual processing of the straight-ahead direction? A walking observer is gazing at a flower surrounded by 2 rocks (left panel). One of them is located straight-ahead (SA), while the other one is positioned eccentrically (EC). In such a configuration, the flower forms an image at the center of the retina and is processed optimally by central vision, while both rocks form images in eccentric retinal locations and are processed more coarsely in peripheral vision (right panel, upper row). If the straight-ahead direction receives a privileged processing, the rock that the observer is facing (SA) will receive a prioritized visual processing compared to the rock located eccentrically (EC; right panel, lower row)

Vision is an essential modality in our everyday life that allows natural behaviors such as navigation, exploration of the environment, manipulation of objects… The visual signals captured by our retinas are processed by a dense set of interconnected cortical areas which, in their vast majority, are retinotopically organised (Wandell et al. 2007). Neurons close to one another have receptive fields (RFs) which lie at nearby locations in the image. However, this common retinotopic frame of reference cannot be used as such for efficiently interacting with the three-dimensional environment. It has to be combined with signals from other sensory modalities captured in different (head, body) reference frames. How multi-sensory and sensory-motor spatial coordinate transformations are mediated in the nervous system, especially during ocular explorations, has been the topic of multiple studies in both human and non-human primates over the last decades. The pioneer electrophysiological works by V. Mountcastle and colleagues showed modulations of visual responses by gaze direction^1^ in the macaque associative parietal cortex. These were described as gain modulation in which intrinsic properties of the visual neurons remain unchanged but the overall spike rate depends on the monkey gaze direction in space (Andersen and Mountcastle 1983; Andersen et al. 1985). This integrative process was subsequently shown to arise as early as the primary visual (V1) area and to extend through extrastriate areas and up to the premotor cortex (Trotter and Celebrini 1999; Boussaoud and Bremmer 1999). These gain modulations were then confirmed in humans from fMRI experiments which showed that gaze direction can be recovered from multivariate analyses of BOLD responses measured in area V1 (Merriam et al. 2013). These findings have considerably challenged our understanding of the functions performed by the primate early visual cortex which would not only reflect processes in a retinotopic coordinate system but could actually be involved in coordinate transformations. At the theoretical level, the possibility to recover the egocentric location of visual elements through a set of gain-modulated visual neurons has been demonstrated from studies in computational neurosciences (Zypser and Andersen 1988; Pouget and Sejnowski 1997; Salinas and Sejnowski 2001). Interestingly, most of these models assumed that all egocentric positions (i.e., combinations between receptive fields and gaze directions) are similarly represented and distributed in cortical areas that combine these signals (Andersen 1990; Bremmer et al 1998). Ten years ago, a study from our group in the macaque showed that it is not always the case. We found that most neurons coding for peripheral vision have stronger firing rates when gaze direction brings their receptive fields along the straight-ahead direction. (Durand et al. 2010). This particular axis is ecologically very relevant for spatial cognition, for example in the case of collision avoidance during navigation (see Fig. 1).

Therefore, cortical gain modulations may not be restricted to only coordinate transformations and may also support other important cognitive functions. Over the last decade, several studies have confirmed this finding of a privileged processing of the straight-ahead direction and documented it at different levels. Here, we review the results of these works. We start with the description of the original monkey electrophysiology recordings that demonstrated a consistent increase in the neural responses to stimuli located straight-ahead the head/body midline ("First findings in electrophysiology" ). We subsequently present studies performed in humans using cerebral recordings (fMRI and EEG) which found that straight-ahead effects are observable at the macroscopic level and in other primate species ("Effects at the macroscopic level"). We then show that this privileged neural encoding of the straight-ahead direction impacts visual perception and oculomotor behaviour ("Behavioural effects"). Finally, we discuss the implications of all these findings for visual and spatial cognition in primates and present what we believe to be important leads for future studies on this fascinating topic.

### First findings in electrophysiology

We saw in the introduction that modulations of neural excitability triggered by changes in gaze direction are ubiquitous in the primate visual cortex and can be observed as early as primary area V1. The demonstration was notably brought by Trotter and Celebrini (1999), who performed extracellular single-unit recordings in the dorsal part of V1 in behaving macaque monkeys trained to maintain fixation at various gaze angles. After isolating the spiking activity of single neurons under the tip of the microelectrode, their receptive fields (RF) were characterized and subsequently stimulated with luminance square-wave gratings and dynamic random dot stereograms. Neural responses to the same visual stimulations were recorded, while the animals were instructed to gaze in different directions. In most recorded neurons (~ 70%), the spiking activity was found to vary significantly as a function of gaze direction. Importantly, changes in the direction of gaze mostly affected the overall level of the neuron’s excitability (i.e., their gain), but only seldomly their tuning for orientation and binocular disparity.

**Fig. 2:**
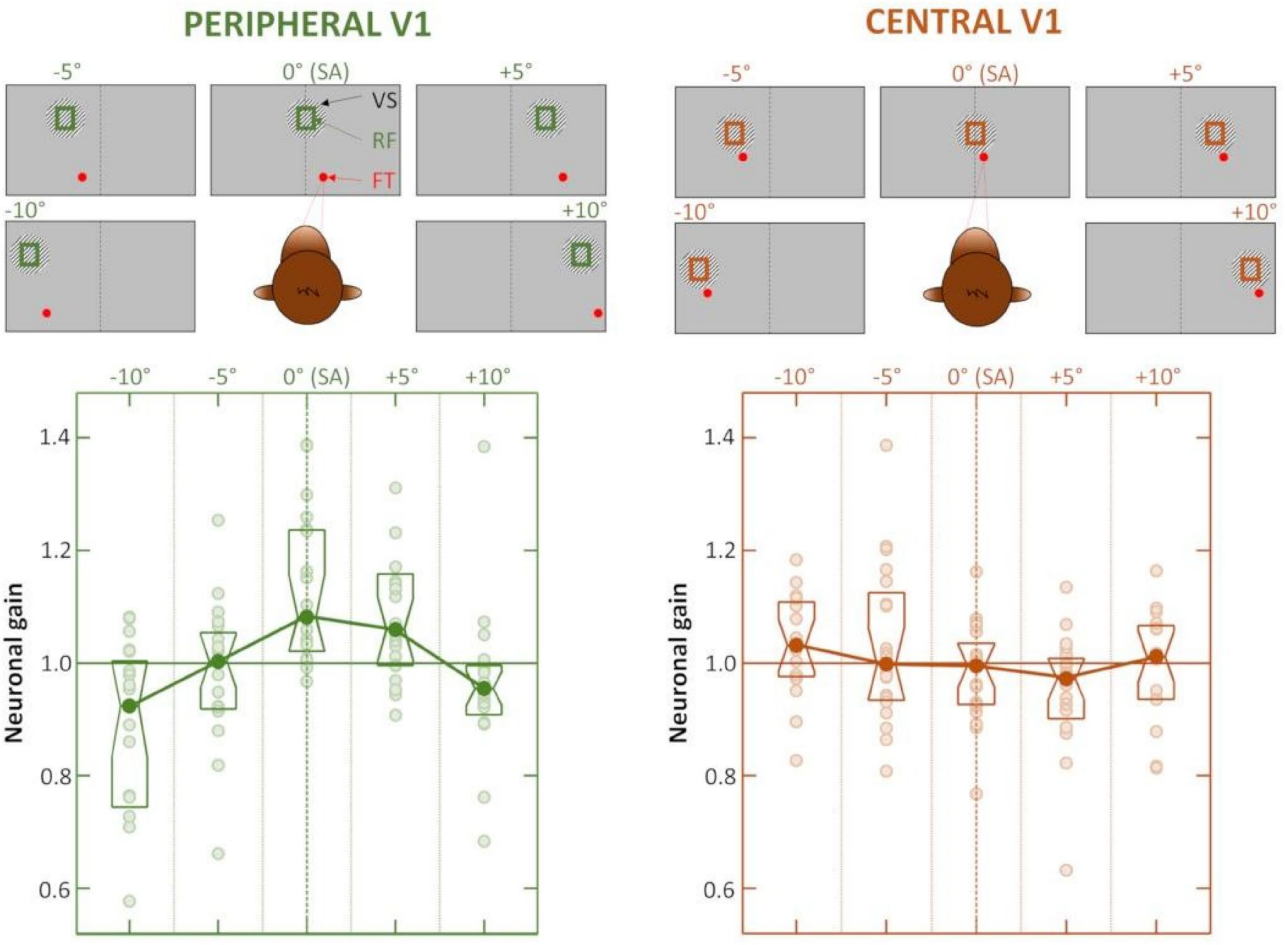
Privileged processing of the straight-ahead direction in macaque peripheral area V1. Multi-unit recordings in V1 targeted neurons with population receptive fields (RF) in the peripheral (left, in green) or central (right, in orange) portions of the visual field. The monkey maintained its gaze on a fixation target (FT) whose position was adjusted to move the pRF of the recorded units between − 10° and + 10° with steps of 5°. The pRF always received the same visual stimulations (VS), which were either dynamic random dot stereograms or luminance gratings. Modulations of multi-unit activity by gaze direction were observed in both peripheral and central V1. However, only peripheral V1 showed a significant increase in neuronal gain at the population level when the pRF are aligned with the straight-ahead (SA) direction. Figure adapted from Durand et al. (2010)

Neurons in the dorsal part of area V1 have small RFs subtending a few degrees around the fixation target (retinal eccentricity less than 5 degrees). They are in charge of central vision. Some years later, the same team (Durand et al. 2010) used a similar approach to investigate the effects of gaze direction on V1 neurons lying within the calcarine sulcus and in charge of the contralateral peripheral visual field (retinal eccentricity between 7 and 35 degrees). Gain modulations associated with the direction of gaze were found to be as frequent in peripheral V1 as in central V1 (~ 70% of the recorded neurons). However, a notable difference was that most peripheral V1 neurons exhibited their maximal gain for the gaze direction bringing their RF along the straight-ahead direction. To confirm this observation, multi-unit recordings were performed in both central and peripheral V1 of the same animal. Usually, both aspects of area V1 were tested within the same recording sessions, since the penetration angle of the microelectrode was set to encounter first the dorsal part of V1, and then its deeper calcarine sector. Once the population RF of a recording site was localized, 5 directions of gaze bringing the RF between 10° left and 10° right, with 5° steps were successively tested (Fig. 2, upper line). Across the multi-unit recording sites, gain modulations associated with changes in gaze direction were usually weaker in central than in peripheral V1, indicating that gaze direction preferences are probably more balanced across neighbouring central V1 neurons and tend to cancel out when averaged together (Fig. 2, lower-right panel). In peripheral V1, the bias for the straight-ahead direction was fully confirmed, with the population gain profile showing an inverted ‘V’ profile peaking at the straight-ahead direction (Fig. 2, lower-left panel). Further control experiments showed that this effect could not be accounted for by slight gaze-dependent variations in the luminance, position, size or binocular disparity of the visual stimulation. Thus, such a gain profile indicates that at the population level, peripheral V1 neurons are maximally sensitive to visual stimuli located straight-ahead. Although the straight-ahead preference was not observed in the spontaneous activity of V1 neurons, it was already present in the earliest components of their visual responses (at about 40 ms post stimulus onset). A subsequent study reported that a neuronal preference for the straight-ahead direction might also be present across central V1 neurons (Przybyszewski et al. 2014). This apparent discrepancy between the results of Durand et al. (2010) and those of Przybyszewski et al. (2014) concerning central V1 might be due to differences in the protocols used in both studies. The latter used bright or dark bars optimized in terms of orientation, length, velocity and color for the recorded neurons so as to measure their contrast response functions. The gain effect that was reported was significant only for the dark bars and statistically weaker at the population level. It would be worthwhile to confirm the presence of a straight-ahead bias in central V1 through multi-unit recordings. At this stage, one can only postulate that if this bias exists, it is less strong and systematic (in terms of stimuli parameters) than that observed in peripheral V1.

These results are important for two main reasons. First, they show that gaze-dependent gain modulations do not necessarily average out at the population level and thus, that they could have a significant influence on the overall visual sensitivity per se. Second, they reveal that a greater visual sensitivity is allocated to the objects we are facing, which probably reflect their special behavioural status, notably because they represent potential obstacles during locomotion. If this mechanism is present in the visual cortex of macaque monkeys, an important question is whether it can also be found in that of humans. The following section provides evidence gathered through non-invasive macroscopic approaches (fMRI and EEG) that this is actually the case.

### Effects at the macroscopic level

**Fig. 3:**
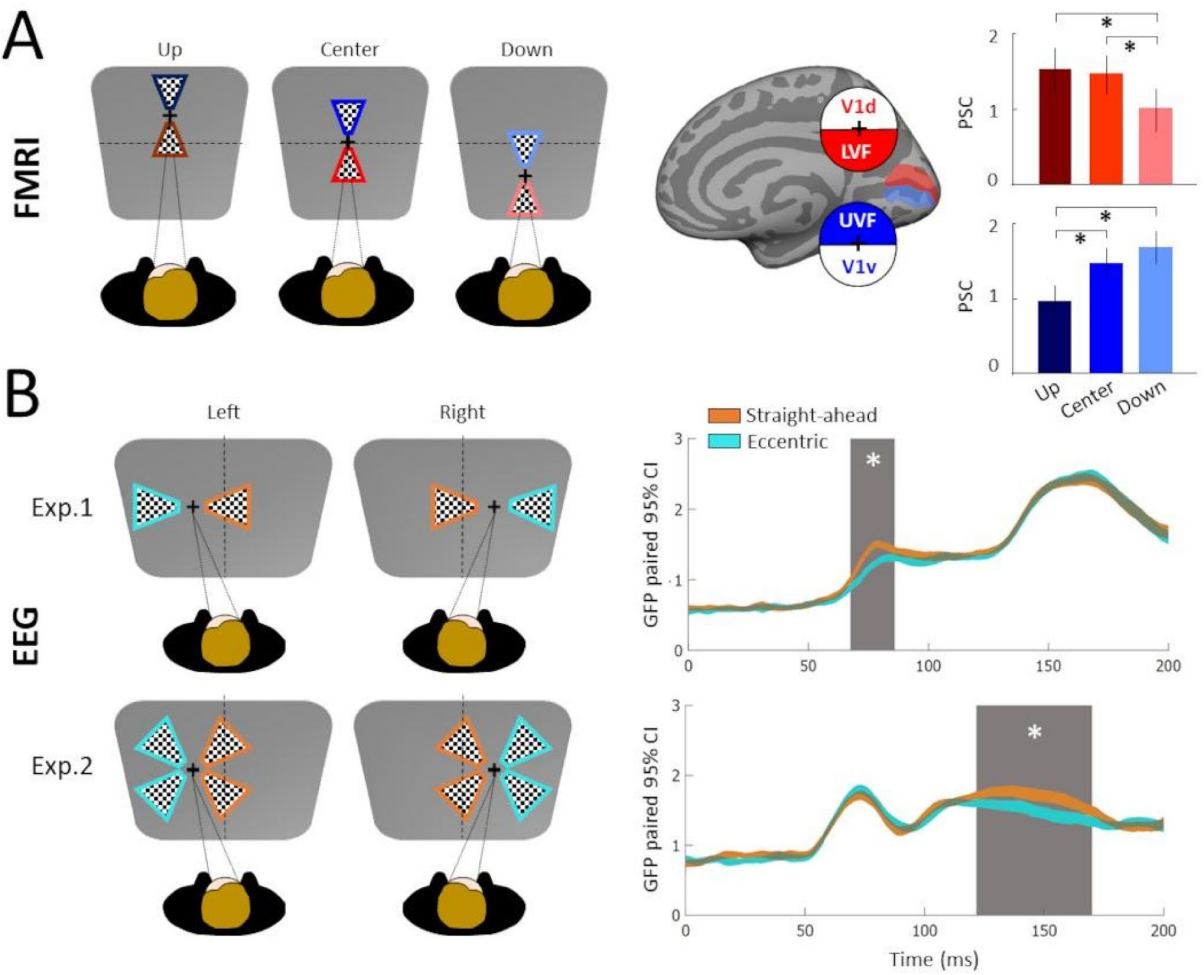
Macroscopic evidence of a privileged processing of the straight-ahead direction in the human visual cortex. **A** fMRI design and results. Participants (*n* = 6) were gazing 20° up, at eye level, or 20° down while exposed to rotating checkerboard wedges. Average BOLD responses (in percent signal change, PSC) across the dorsal (V1d, in red) and ventral (V1v, in blue) parts of V1, housing the representations of the lower visual field (LVF) and upper visual field (UVF), respectively. In dorsal V1, responses to the stimulation along the lower vertical meridian increase as the gaze goes up, while in ventral V1, responses to the stimulation of the upper vertical meridian increase as the gaze goes down. In both cases, maximal responses are observed for stimulation along the straight-ahead direction. Figure adapted from Strappini et al. (2015). **B** EEG design and results. Participants (*n* = 29) were gazing 10° left or 10° right while exposed to checkerboard wedges. In the first experiment, these wedges were presented along the horizontal meridians, either on the left or on the right (upper-left panel). The Global Field Powers (GFPs) corresponding to the grand average differential ERPs for straight-ahead (orange) and eccentric (pale green) stimulations (see details in the text) are shown in the upper-right panel. The width of these time-courses provide their 95% confidence intervals. The shaded area corresponds to a time-window during which the two time-courses are significantly different. Straight-ahead effects can be observed as early as 70 ms after stimulus onset. In the second experiment, the checkerboard wedges were presented on the diagonals, in one of the four visual quadrants (lower-left panel). The GFPs of the differential ERPs for straight-ahead (orange) and eccentric (pale green) only differ around 130 ms after stimulus onset but not for the C1 component (see the first peak at *t* = 75 ms)(lower-right panel). Figure adapted from Bogdanova et al. (2020)

In this section, we present two studies that investigated whether the straight-ahead direction is also associated with privileged processing in the human visual system using macroscopic recordings (fMRI and EEG). The first study to test whether the straight-ahead direction modulates responses in the human visual cortex was based on fMRI recordings and conducted by Strappini and collaborators (2015). These authors combined standard phase-encoded stimuli (flickering checkerboards in either a wedge or a ring configuration for polar angle and eccentricity mapping, see, e.g., Sereno et al. 1995) and wide-field stimulation (see Pitzalis et al. 2006) to characterize the retinotopic properties within the visual cortex of 6 participants. By determining the preferred polar angle and eccentricity of each visually responsive voxel from an analysis in the Fourier domain, this approach permitted to delineate the borders of several retinotopic areas (notably V1, V2, V3, V4 and V3A) and also to define subregions of V1 and V2 responding to stimulations along the horizontal or vertical meridians. In the same group of subjects, they subsequently performed recordings to test for the interaction between retinotopy and gaze direction. They used a 10° radius flickering checkerboard wedge rotating in a counter-clockwise direction around an ocular fixation point located either straight-ahead (gaze center condition) or 20° up (gaze-up) or down (gaze-down), along the vertical meridian (see Fig. 3A, upper panel). They found that although gaze direction did not modify the preferred polar angle of the responsive voxels (see supplementary figure 9 in Strappini et al. 2015), it could nevertheless modulate their response gain. This is in agreement with the results of a previous fMRI study that investigated the relationship between gaze direction and retinotopic properties (Merriam et al. 2013). Gain modulations were nearly absent in the subregions of V1 and V2 coding for the horizontal meridian, while they were strong and consistent in subregions coding for the vertical meridian. By examining in more details the response profiles in the dorsal and ventral part of these subregions of V1 and V2, they found that in dorsal V1 (V1d) and dorsal V2 (V2d), response amplitudes to stimulation along the lower vertical meridian were significantly stronger (BOLD responses were up to 25% more important) for the gaze-center and gaze-up conditions when compared to those of the gaze-down condition. At the opposite, in ventral V1 (V1v) and ventral V2 (V2v), response amplitudes to stimulation along the upper vertical meridian were significantly stronger for the gaze-center and gaze-down conditions when compared to those of the gaze-up condition (see Fig. 3A, lower panel for results in V1v and V1d). The lower responses observed in V1 and V2 when stimuli are localized more eccentrically with respect to the eye level demonstrate a preference for straight-ahead stimulation in these two areas.

The privileged processing of the straight-ahead direction that was first demonstrated in macaque V1 using single-cell and multi-unit recordings (Durand et al. 2010, see the previous section) thus generalizes to macroscopic recordings in humans. Interestingly, the strength of the gain modulations reported in humans (i.e., an improvement of the BOLD responses by as much as 25% for straight-ahead stimuli) is quite consistent with the spike-rate increases observed in macaques (i.e., about 20–40% when gaze direction puts the receptive field straight-ahead the head/body midline), which suggests similar processes of the straight-ahead direction in the two primate species. Finally, it seems important to point out that gaze direction in the fMRI study was distributed along the vertical meridian, whereas it was distributed along the horizontal meridian in the electrophysiological study. It suggests that the privileged processing of the straight-ahead direction in the visual system of human and non-human primates is operant along both the horizontal and vertical axes of the egocentric space. This hypothesis is in line with the fact that gain modulations were reported for gaze direction along both the horizontal and vertical direction. For example, in their human fMRI study, Merriam et al. (2013) were able to decode between 8 different eccentric eye positions (either along cardinal directions or on the diagonals) from response amplitudes in several visual areas, and notably V1. Note, however, that this study did not explore whether specific spatial positions (e.g., the straight-ahead direction) were systematically associated with gain increases.

If the spatial resolution of fMRI is good enough to determine that straight-ahead effects occur in early visual areas, and notably in V1 (Strappini et al. 2015), its temporal resolution remains too poor to capture the dynamics of the underlying mechanisms. To characterize these dynamics, Bogdanova et al. (2020) performed EEG recordings in human participants. Their stimulus was a flickering checkerboard wedge at high contrast that was presented along the horizontal meridian (i.e., at eye level) at 10° of eccentricity either to the left or to the right of ocular fixation. As shown in Fig. 3B (upper panel), gaze direction was also manipulated along the horizontal meridian so that during leftward gaze (− 10°), visual stimulations in the right visual field were straight-ahead the head/body midline, whereas visual stimulations in the left visual field were eccentric (i.e., at − 20° with respect to the straight-ahead direction). At the opposite, during rightward gaze (+ 10°), visual stimulations in the left visual field were straight-ahead, whereas visual stimulations in the right visual field were eccentric. The authors first compared the event-related potentials (ERPs) in response to straight-ahead vs eccentric stimulations and found that, although retinal inputs were identical in the two conditions, peak amplitudes of the P1 bilateral (~ 140 ms after stimulus onset), N1 bilateral (~ 200 ms after stimulus onset) and P2–P3 (~ 300 ms after stimulus onset) components were significantly higher for straight-ahead stimuli (see their Fig. 2). Peak latencies did not differ between the two conditions. These effects are consistent with a gain modulation of the responses, in line with the previous findings in macaque electrophysiology (see Sect. "First findings in electrophysiology") and human fMRI (see above).

To determine the first occurence of these straight-ahead effects in their data, Bogdanova et al. (2020) subsequently computed the difference between EEG responses evoked by stimulations in the left and right visual hemi-fields. This operation permits to highlight another ERP component, the P1 contralateral, which peaks around 80 ms after stimulus onset (see Fig. 3B, upper panel) and which is believed to reflect responses from extrastriate visual areas V3, V3A and V4 (Di Russo et al. 2002). While the latency of the P1 contralateral component was independent of the condition, its amplitude was significantly more important for straight-ahead stimuli, as early as 65–70 ms after stimulus onset (see the global field powers in the upper-right panel of Fig. 3B.

To go further along this direction, the authors performed a second experiment which was specifically designed to test whether the straight-ahead direction also affects the earliest observable visual ERPs. It was based on the same experimental design except that the flickering checkerboard wedge was randomly presented at 10° of eccentricity within one of the four visual quadrants (Fig. 3B, lower-left panel). Bogdanova and colleagues subsequently computed the difference between EEG responses to stimulations in the upper vs lower visual field. This operation permits defining the C1 component which, respectively, arises and peaks around 55 and 75 ms after stimulus onset and which is believed to reflect the first feedforward visual processes in early visual areas (i.e., in V1, V2 and V3, see Ales et al. 2010). The peaks and latencies of this C1 component were not significantly different for straight-ahead vs eccentric stimulations (see Fig. 3B, lower-right panel). Stronger amplitudes for straight-ahead stimulations only appeared later, notably for the P1 bilateral, N1 bilateral and P2–P3 components, in agreement with the results of the first experiment. Finally, it is also worth reporting that in these two EEG experiments, power in the alpha band before stimulus onset was dependent on eye position: it was reduced in the hemisphere ipsilateral to gaze direction and thus contralateral to the straight-ahead direction. This finding is in line with previous EEG studies which showed that pre-stimulus alpha power suppression is associated with enhanced evoked activity (Van Dijk et al. 2008) and modifications of the P1 amplitude (Fellinger et al. 2011). It suggests that the privileged processing of the straight-ahead direction could be associated with a modification of the spontaneous activity that emerges as early as the eyes move toward an eccentric position.

Altogether, these two studies demonstrated that straight-ahead effects are also observable in the human occipital cortex (notably in area V1, Strappini et al. 2015) as early as 65–70 ms after stimulus onset (Bogdanova et al. 2020). If these results are generally compatible with those observed in monkey electrophysiology, (Durand et al. 2010, see Sect. "First findings in electrophysiology"), further studies will be necessary to determine whether the earlier effects observed in macaque (i.e., straight-ahead facilitations appear at about 40 ms after stimulus onset in this species) reflect a methodological (single/multi-unit recordings vs scalp EEG) and/or a species difference. The recent development of techniques such as intracranial recordings in implanted epileptic patients (De Jong et al. 2016) and/or monkey EEG (Sandhaeger et al. 2019) could permit to address this issue by making it possible to compare the effects obtained in the two species from the same methodological approach.

### Behavioural effects

Studies in human (fMRI and EEG, see "Effects at the macroscopic level") and non-human (single and multi-unit recordings in macaque, see "First findings in electrophysiology") primates showed consistent increases in the neural responses to straight-ahead stimuli. What is the purpose of these specific gain modulations? In this section, we describe two studies that investigated behavioural consequences of this privileged visual processing of the straight-ahead direction.

**Fig. 4:**
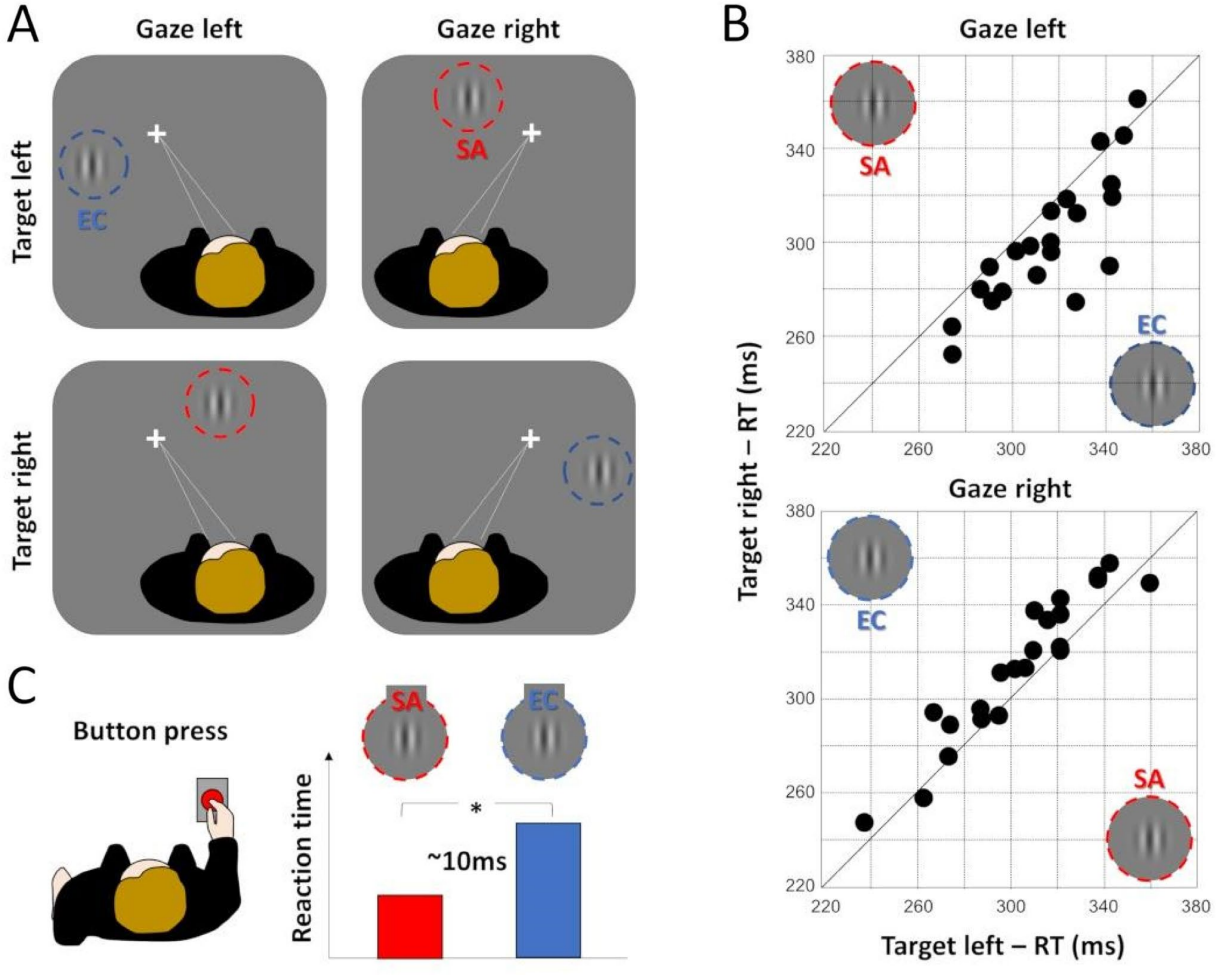
Behavioural evidence of a privileged processing of the straight-ahead direction. **A** Experimental design. Participants (*n* = 20) were involved in a simple reaction time task. They had to press a button as quickly as possible after the appearance of a peripheral visual target. During the task, participants maintained their gaze 10° left or 10° right, while peripheral visual targets were flashed pseudo-randomly 10° left or 10° right with respect to the center of gaze along the horizontal meridian. This produces a 2*2 factorial design with 2 straight-ahead (SA) targets and 2 eccentric (EC) targets. **B** Individual reaction times. Mean participants’ reaction times for the left vs right targets with the gaze left (upper pane) and the gaze right (lower panel). In both cases, most participants showed shorter reaction times for the SA targets. **C** Overall results. On average, behavioural responses for the SA targets were about 10 ms (ms) shorter than for the EC targets. Figure adapted from Durand et al. (2012)

In a first study, Durand et al. (2012) used a visual detection task to examine whether objects presented in the peripheral field of view elicit faster reaction times (RTs) when they are located straight-ahead rather than eccentric with respect to the head/body midline. Their stimulus was a vertically oriented Gabor patch displayed along the horizontal meridian. They used a 2 by 2 factorial design with retinotopic position (± 10°) and gaze direction (10° leftward or rightward) as main factors (see Fig. 4A). Participants (*n* = 21) were instructed to maintain their gaze on a central fixation cross and to detect and report as quickly as possible the brief apparitions of the Gabor patch with a button press. Importantly, the orientation of the screen was adapted for leftward and rightward fixation so that it remains perpendicular to the gaze axis (see their Fig. 2). This permitted to equalize the distances of all the targets from the observers. The analyses of the data revealed that during leftward gaze, median RTs were shorter for targets presented in the right visual field for 19 out of 21 participants. On the other hand, during rightward gaze, median RTs were shorter for targets presented in the left visual field for 18 out of 21 participants (see Fig. 4B). A statistical analysis based on a two-way RM ANOVA confirmed that neither retinotopic position nor gaze direction had a significant impact on RTs. The interaction term was nonetheless highly significant, revealing a clear effect of the target’s location with respect to the head/body axis. Across all the participants, median RTs were found to be about 10 ms shorter for straight-ahead vs for eccentric targets (Fig. 4C).

Importantly, gaze direction was recorded with an eye-tracker in all the participants and the analyses of these oculomotor data confirmed that the results were not driven by biases in ocular fixation (see Fig. 4 in Durand et al. 2012).

In their study, Durand et al. (2012) performed three important additional experiments. They first showed that their results remained unchanged under monocular viewing, as was also observed in monkey electrophysiology (see Durand et al. 2010). It demonstrates that the shorter RTs observed for straight-ahead stimuli are not caused by differences between the binocular (notably vertical) disparities contained in the two experimental conditions. In a second control experiment, they showed that straight-ahead facilitations were still observable when the subjects were involved in a dual task (to detect brief dimming of the fixation point). It suggests that the privileged processing of the straight-ahead direction does not require full attentional resources. We will further comment on the involvement of attention in straight-ahead facilitations in the discussion. Finally, in a third control experiment, participants were involved in the same dual task but the Gabor patches were presented along the upper vertical meridian (10° above the fixation point) and gaze direction was manipulated parametrically (− 10°, − 5°, 0°, 5° and 10°). Shorter RTs were always observed for Gabor patches presented straight-ahead (i.e., when ocular fixation was at 0°). It demonstrates that the straight-ahead preference is not caused by objects presented contralaterally to gaze direction. It also shows that this effect can be generalized to stimuli presented along the vertical meridian, in line with the neuroimaging (fMRI) finding described in Sect. "Effects at the macroscopic level" (Strappini et al. 2015).

**Fig. 5:**
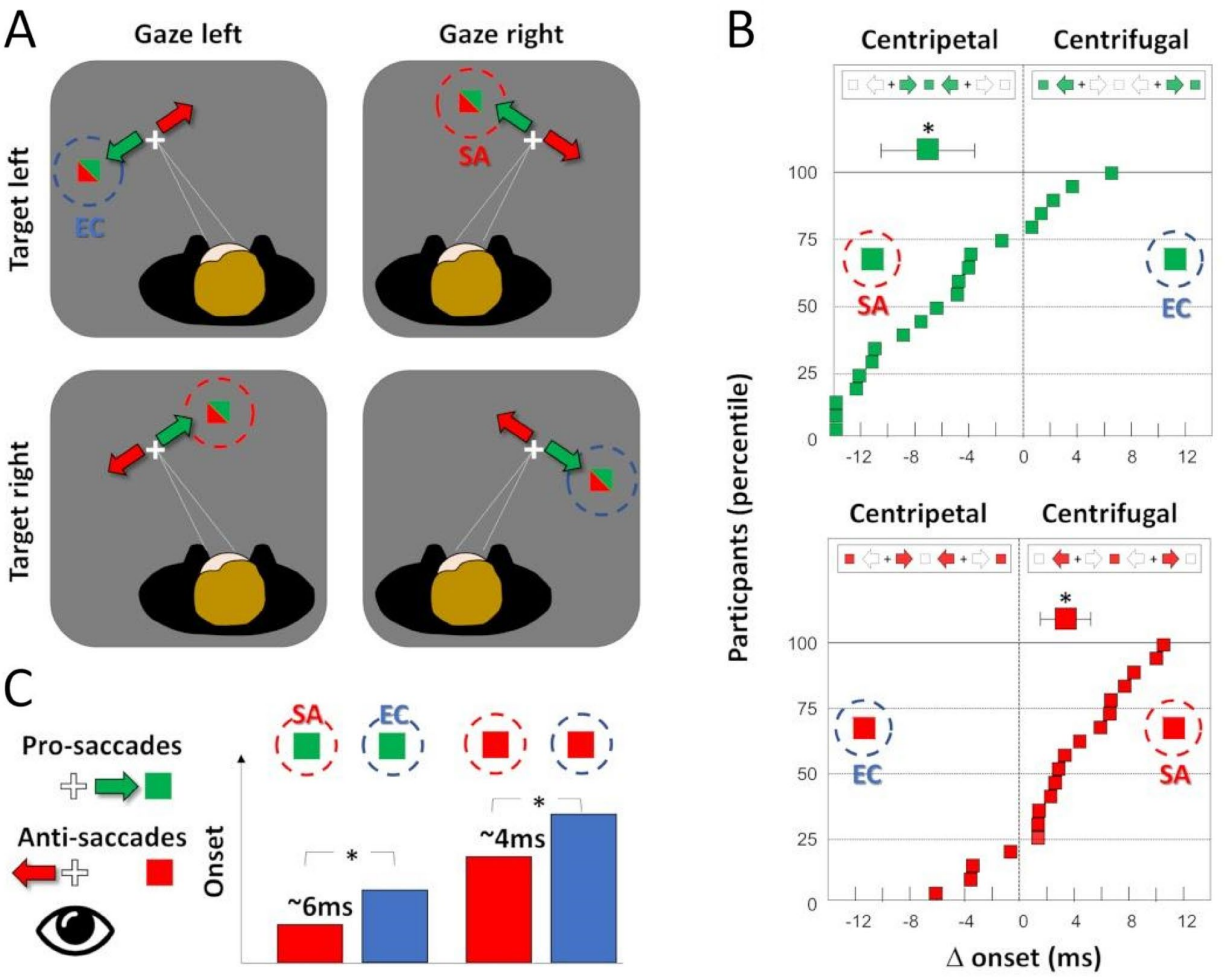
Oculomotor evidence of a privileged processing of the straight-ahead direction. **A** Experimental design. Participants *n* = 20 were involved in pro- and anti-saccade tasks. They had to move the eyes toward (pro-saccades) or away from (anti-saccades) a peripheral visual target (green targets for pro-saccades and red targets for anti-saccades). During the task, participants had their gaze 10° left or 10° right, while peripheral visual targets were flashed pseudo-randomly 10° left or 10° right with respect to the center of gaze along the horizontal meridian. This produces a 2*2 factorial design with 2 straight-ahead (SA) targets and 2 eccentric (EC) targets. **B** Individual differences in onset times between centripetal and centrifugal saccades. Mean participant’s differences in onset times for centripetal and centrifugal saccades for the pro-saccades (upper panel) and the anti-saccades (lower panel). In both cases, most participants showed shorter onset times for the SA targets. **C** Overall results. On average, for both pro- and anti-saccades, those triggered by SA targets had shorter onsets than those triggered by EC targets. Figure adapted from Camors et al. (2016)

In a following study, Durand and colleagues (Camors et al. 2016) addressed the potential impact of the privileged visual processing of the straight-ahead direction on oculomotor behaviour. Saccadic eye movements bringing the eyes toward the straight-ahead direction (centripetal saccades) are known to be initiated faster (Paré and Munoz 2001) and to reach higher peak velocity (Pelisson and Brablanc 1988) than saccades steering the eyes away from that direction (centrifugal saccades). However, it was generally admitted that the faster dynamics of centripetal saccades had motor origins, either linked to their privileged preparation and/or easiest execution. To investigate the sensory impact of presenting a saccadic target along or away from the straight-ahead direction, the authors used both pro- and anti-saccadic tasks: participants were instructed to move the eyes either toward or away from a suddenly appearing peripheral visual target. Such a design (Fig. 5A) allows dissociating the motor influence of the saccade’s direction (centripetal vs centrifugal) and the sensory influence of the visual target’s location (straight-ahead vs eccentric), since both centripetal and centrifugal saccades could be elicited by straight-ahead or eccentric targets. Centripetal pro-saccades were initiated earlier than centrifugal ones (see Fig. 5B, upper panel), as previously described by others (Laurutis and Robinson 1986). Importantly, results were reversed for anti-saccades (Fig. 5B, lower panel): centripetal anti-saccades were initiated later than centrifugal ones. What centripetal pro-saccades and centrifugal anti-saccades have in common is the fact that they were both triggered by straight-ahead targets, thus revealing that their faster initiation has a sensory rather than a motor origin. Interestingly, in both the pro- and anti-saccade tasks, centripetal saccades always reached higher peak velocity than centrifugal ones, ruling out a sensory origin for the faster execution. Overall, these results indicate that both sensory and motor factors contribute to the superior temporal properties of centripetal saccades: they are executed faster because of facilitated motor preparation/execution, but their faster initiation is explained by the privileged visual processing of visual elements located straight-ahead.

Interestingly, the execution of centripetal and centrifugal saccades has been shown to evoke differential neural activity in the superior colliculus of both monkeys (Paré and Munoz 2001) and humans (Krebs et al. 2010). Yet, it would be hazardous to make a link with the visual straight-ahead bias described here. In monkeys, modulations of collicular activity precede the appearance of the visual target and they only concern the fast saccades. In humans, reduced BOLD activations for centripetal saccades are observed in the colliculus, but not in the parietal and frontal eye fields. Although these results suggest that the observed modulations have an oculomotor rather than a visual origin, we have shown the necessity to introduce both pro- and anti-saccade tasks to disentangle the respective contributions of both components.

## Discussion

Gaze direction has been shown to exert a strong and widespread influence in the visual cortex of human and non-human primates. However, this influence has generally been considered as reflecting exclusively spatial processes leading to reference frame transformations for multisensory integration and sensorimotor control. This influence was tacitly thought to have no macroscopic effect on visual processing per se. Individually, most visual neurons are gain-modulated by changing the direction of gaze, but at the population level, these individual modulations cancel out due to the diversity of gain-field profiles across neighbouring neurons. Here, we have reviewed studies that demonstrated a consistent influence of gaze direction on visual processing at different levels (single neurons, neural populations, cortical areas and behaviour) and in different primate species (humans and macaques). Most neurons in charge of the peripheral field of view exhibit their maximal gain for gaze direction aligning their receptive field with the straight-ahead direction. As a consequence, straight-ahead stimuli are associated with stronger responses in individual neurons and neural populations ("First findings in electrophysiology") and also in macroscopic measurements ("Effects at the macroscopic level"). They also impact visual perception and oculomotor behaviour as they are detected earlier and trigger saccades with earlier onsets ("Behavioural effects"). We further discuss here the cognitive functions of these straight-ahead modulations and their neural substrates. We also suggest potential leads for future studies on this topic.

### Locations and nature of the involved modulatory signals

If the studies described in this review focused on area V1, it is likely that several other visual areas are also engaged in the privileged processing of the straight-ahead direction. Indeed, the human fMRI recordings (Strappini et al. 2015, see "Behavioural effects") showed strong straight-ahead effects in V2. The same study found significant interactions between visual stimulation and eye position in several retinotopic areas which suggests their possible implication in egocentric processing, in line with previous fMRI results (Deutschländer et al. 2005). In EEG, the straight-ahead direction is associated with a stronger amplitude of the contralateral P1 component (Bogdanova et al. 2020), which is believed to reflect neural responses from mid-level visual areas V3, V3A and V4 (Di Russo et al. 2002). It is, therefore, likely that the privileged processing of straight-ahead stimuli is ubiquitous in the primate visual system, which raises the question of how and where it emerges. Classically, it is hypothesized that gaze direction modulations are mediated by different extraretinal signals either proprioceptive, or efference copy (corollary discharge) or both. Indeed, early visual cortex (and notably area V1, where straight-ahead effects were first measured) receives proprioceptive projections whose implications for spatial cognition are well documented, notably by developmental studies (Steinbach 1987; Buisseret 1995; Donaldson 2000 for reviews). Its activity is also influenced by the efference copy, especially during eye movements, to account for visual stability (Morris and Krekelberg 2019; Wurtz 2008 for review). In addition, a visual signal, the vertical binocular disparities that exist at peripheral eccentricities, could also be used to recover the 3-D space characteristics with or without an extraretinal source of information on the position of the eyes (Mayhew and Longuet Higgins 1982; Garding et al. 1995). If vertical disparities are actually encoded in V1 (Durand et al. 2002, 2007), their involvement in straight-ahead modulations was discarded as these modulations persist under monocular viewing (Durand et al. 2010). The origin of the straight ahead modulations might be accounted for by the early integration of these extra-retinal signals (proprioception, efference copies) with visual inputs in primary visual cortex and even subcortical areas like the LGN (Donalson and Dixon 1980; Lal and Friedlander 1989), where a neural mechanism for localization of visual targets would exist, leading to a possible feedforward propagation of the straight-ahead preference. Alternatively, this preference might emerge in higher order associative areas, with a feedback propagation toward the early visual cortex that would raise when gaze direction is changed.

Whatever the feedforward or feedback nature of the propagation of the straight-ahead preference, this phenomenon can be considered as a mechanism of spatial attention: it serves allocating more neuronal resources for the visual analysis of a behaviourally relevant portion of the surrounding space. In line with this idea, the neural gain increases measured for straight-ahead stimuli in primary visual cortex (Strappini et al. 2015) and at early latencies (Bogdanova et al. 2020) are similar to those observed when manipulating attention (see, e.g., Kastner et al. 1999 or Liu et al. 2005 for fMRI studies and Hillyard and Anllo-Vento 1998 for EEG findings). Classically, spatial attention mechanisms fall into one of 2 classes. Endogenous attention refers to internal mechanisms in which attention is deployed voluntarily toward a portion of space selected on the basis of prior knowledge and cognitive operations (such as task demand, probability of relevance, etc.). As such, it is considered as a top-down (or feedback) mechanism. Exogenous attention, by contrast, is deployed reflexively toward locations in which unexpected and salient external events occur. Exogenous attention is generally seen as a bottom-up (feedforward) mechanism. Interestingly, the gain increases for straight-ahead stimuli described in the present review fail to completely fit within either of the 2 classes but rather, they share some characteristics with both of them. Because the signals associated with eye position (be it proprioceptive or efference copy, see above) are internal and trigger a privileged processing of a relevant portion of the surrounding space, straight-ahead modulations can be seen as endogenous. On the other hand, straight-ahead effects can be related to exogenous attention, as they are processed involuntarily and regardless of the ongoing task (see, e.g., Corbetta and Shulman 2002 for review). Indeed, in the fMRI study of Strappini and colleagues (2015), participants were either instructed to passively view the stimulus or to perform a task designed to strengthen covert visual attention on the stimulus (they had to detect the rare appearance of a letter within a continuous stream of letters superimposed on the rotating wedge). This manipulation did not affect the results as significant straight-ahead effects were observed in both cases. In our behavioural study (Durand et al. 2012), shorter reaction times for straight-ahead stimuli were maintained when participants performed a dual task (i.e., to detect brief dimming of the fixation point). These results demonstrate that straight-ahead effects remain significant even when attention is deviated toward another task. In the end, the straight-ahead effect could actually rely on a third class of attention mechanisms that share properties with both endogenous and exogenous attention, i.e., the experience dependent control of attention or history-driven selection as recently proposed by several studies (Theeuwes 2018, 2019; Chelazzi and Santandrea 2018). In that scheme, visual attention can be shaped by previous interactions of the individual with the environment through various forms of experience-dependent plasticity prioritizing the processing of specific spatial locations. This history-based selection has been shown to establish relatively quickly in various contexts including reward, statistical learning, priming and acquired fear. Fundamental learning processes influence what aspects of the environment stand out to guide attention and action in service of the ultimate goal of survival (Todd and Manaligod 2018 for review). Experience might be effective in inducing plasticity in the brain so as to shape the basic architecture of visual processing with multiple priority maps favoring objects and locations that are relevant for motor (including oculomotor) actions (Chelazzi and Santandrea 2018). Straight-ahead direction is a potential candidate for participating in this mechanism within different areas of the visual hierarchy, starting as early as the striate cortex going through the oculomotor-motor network in an egocentric frame of reference.

### Which part of the egocentric space is privileged?

The experiments described in this review offer ample evidence that when the eyes are directed toward eccentric locations, peripheral vision provides a privileged processing for the visual objects we are facing. However, in all these studies, the head and trunk of the participants were always aligned, implying that the straight-ahead direction was defined similarly by both the head and trunk orientations. What happens when the gaze direction implies both eye and head movements, so that the head and trunk are not aligned anymore? Will peripheral vision allocate more resources to objects located in front of the head, in front of the trunk or in some intermediate directions? This issue actually ties in with the more general question of which part(s) of the egocentric space benefit from privileged visual processing by peripheral vision. The fMRI study of Strappini and colleagues (2015) indicates that along the vertical dimension, the straight-ahead direction seems to be defined by the eyes level, since maximum V1/V2 BOLD responses were observed for checkerboard wedges presented at eyes level (see Fig. 3A). However, the definition of the straight-ahead direction when the head and trunk are not aligned is ambiguous only along the horizontal dimension. Interestingly, in patients suffering from cervical dystonia, a neurological disorder possibly caused by a malfunctioning of the head neural integrator (Shaikh et al. 2016), neither the visual objects aligned with the head or those aligned with the trunk evoked faster reaction times (Amlang et al. 2017). Although this finding does not disentangle whether the impaired preference for straight-ahead stimuli is a cause or the consequence of cervical dystonia, it suggests that normal registration of the head and trunk axes is essential for this phenomenon to occur. To clarify the major anchor of the straight-ahead direction (head-centered and/or trunk-centered), future work should, therefore, also manipulate head orientation.

Our team also established that besides the direction of gaze, the viewing distance could also trigger gain modulations in a large majority of V1 neurons (Trotter et al. 1992, 1996). Such finding was confirmed and extended by other groups (see, e.g., Dobbins et al. 1998; Rosenbluth and Allman 2002). Rosenbluth and Allman (2002) notably combined changes in viewing distance and in gaze direction and confirmed that both parameters strongly impact the gain of most V1, V2 and V4 neurons. Interestingly, Dobbins and colleagues (1998) found that most V1/V2 and V4 neurons also showed a preference for short viewing distances, a finding further confirmed for V4 neurons (Rosenbluth and Allman 2002), but not for V1/V2 (Rosenbluth and Allman 2002; Trotter et al. 1992, 1996). One can note, however, that Trotter and colleagues reported higher rates of spontaneous activity for shorter distances (Trotter et al. 1992, 1996). It remains to be investigated whether neurons in charge of the peripheral visual field could actually exhibit a more pronounced preference for short distances corresponding to the peripersonal space. Such a finding, combined with the straight-ahead preference documented in this review, would suggest that the privileged portion of egocentric space forms a volume located straight-ahead within the peripersonal space. A better characterization of this volume (its properties) constitutes an important lead for future research on this topic.

## Conclusion and directions for future work

Overall, we have reviewed converging evidence suggesting that human and non-human primates are endowed with a mechanism for prioritizing the visual processing of objects lying in front of the body. This prioritisation might echo the behavioural importance of the faced objects, which constitute privileged targets for directed actions or potential obstacles during navigation (especially if they are close to the observer in the peripersonal space). With respect to this point, an important issue will be to assess how this sensory prioritization is repercuted in the visuomotor circuits that control locomotion (Smith et al. 2018; De Castro et al. 2021) and object manipulations (Rizzolatti and Matelli 2003; Tomassini et al. 2007), which appear to be very similar between humans and monkeys. At least one functional consequence of this prioritisation has been established: the possibility for straight-ahead objects to evoke faster behavioural responses. Yet, many aspects of this new egocentric attentional mechanism remain to be clarified. The exact shape of this egocentric spotlight should be defined with more accuracy, in both the horizontal, vertical and depth dimensions. Determining whether this prioritized portion of egocentric space is anchored to the head or to the trunk is also an important issue that will help reach a better understanding of its ecological utility. For instance, one can postulate that if the straight-ahead bias is mostly dedicated to the detection of obstacles during locomotion, then the main anchor should be the trunk and the bias might be even more pronounced during self-displacement. Another alternative might be that this bias is more versatile and can mediate various types of object-directed actions. In that case, the head or trunk anchoring might depend on the actual behavioural context. Whatever the range of actions for which a straight-ahead bias might be useful, another central issue is to know whether responses to straight-ahead objects are only accelerated compared to peripheral objects, or whether detection and/or discrimination thresholds might also be improved, and depend on the behavioural context (i.e., whether straight-ahead objects are potential obstacles, intended targets for reaching/grasping, etc.). Finally, it would be interesting to know whether other sensory modalities also devote special resources for processing the objects we are facing.

## Footnotes

1 In this review, the term ‘gaze direction’ refers to the position of the eyes with respect to the head.
